# Chinese Public’s Engagement in Preventive and Intervening Health Behaviors During the Early Breakout of COVID-19: Cross-Sectional Study

**DOI:** 10.2196/19995

**Published:** 2020-08-21

**Authors:** Zhaomeng Niu, Tingting Wang, Pengwei Hu, Jing Mei, Zhihan Tang

**Affiliations:** 1 Rutgers Cancer Insititute of New Jersey New Brunswick, NJ United States; 2 Sun Yat-Sen University Guangzhou China; 3 IBM Research Beijing China; 4 Hengyang Medical College University of South China Hengyang China

**Keywords:** COVID-19, China, preventive health behaviors, intervening health behaviors, psychosocial, health literacy, behavior, prevention, cross-sectional

## Abstract

**Background:**

Since January 2020, the coronavirus disease (COVID-19) swept over China and then the world, causing a global public health crisis. People’s adoption of preventive and intervening behaviors is critical in curbing the spread of the virus.

**Objective:**

The aim of this study is to evaluate Chinese people’s adoption of health behaviors in responding to COVID-19 and to identify key determinants for their engagement.

**Methods:**

An anonymous online questionnaire was distributed in early February 2020 among Mainland Chinese (18 years or older) to examine their engagement in preventive behaviors (eg, frequent handwashing, wearing masks, staying at home) and intervening behaviors (eg, advising family to wash hands frequently), and to explore potential determinants for their adoption of these health behaviors.

**Results:**

Out of 2949 participants, 55.3% (n=1629) reported frequent engagement in preventive health behaviors, and over 84% (n=2493) performed at least one intervening health behavior. Greater engagement in preventive behaviors was found among participants who received higher education, were married, reported fewer barriers and greater benefits of engagement, reported greater self-efficacy and emotional support, had greater patient-centered communication before, had a greater media literacy level, and had greater new media and traditional media use for COVID-19 news. Greater engagement in intervening behaviors was observed among participants who were married, had lower income, reported greater benefits of health behaviors, had greater patient-centered communication before, had a lower media literacy level, and had a greater new media and traditional media use for COVID-19 news.

**Conclusions:**

Participants’ engagement in coronavirus-related preventive and intervening behaviors was overall high, and the associations varied across demographic and psychosocial variables. Hence, customized health interventions that address the determinants for health behaviors are needed to improve people’s adherence to coronavirus-related behavior guidelines.

## Introduction

In late December 2019, a novel coronavirus disease (COVID-19) emerged in Wuhan, Hubei, China, causing acute pneumonia by the severe acute respiratory syndrome coronavirus 2. COVID-19, being highly infectious and capable of human-to-human transmission, rapidly swept over China (85,921 total confirmed cases in China by July 23, 2020) and the world (15.4 million confirmed cases worldwide by July 23, 2020), developing into a global pandemic [1,2].

The Chinese government promptly implemented nationwide public health emergency measures to control the spread of COVID-19 [3]. Governmental public health strategies have been proven effective in containing infectious diseases [4]. Apart from governmental efforts, the general public plays a crucial role in conquering diseases [5]. There has been compelling evidence that the public’s compliance with precautionary behaviors helps effectively curb the spread of many diseases [5,6]. Thus, it is of great value to evaluate Chinese people’s adoption of health behaviors in responding to COVID-19.

This paper focuses on two distinct types of individual-level health behaviors—preventive health behaviors (PHBs) and intervening health behaviors (IHBs)—in responding to COVID-19. PHB refers to the activities undertaken by a healthy person for the purpose of preventing diseases [7]. In other words, people adopt PHBs to achieve the goal of lessening their own chance of contracting a disease. With the rising number of deaths caused by COVID-19, the Chinese people have significant concerns over COVID-19 for themselves, which would prompt them to adopt PHB. Critically, when facing a public health crisis, people also persuade other people to adopt precautionary behaviors that serve to reduce other people’s risk of contracting the diseases [8,9]. We define the behaviors with a coherent objective of reducing other individuals’ risk to a disease as IHB. As suggested by the definitions, PHB and IHB differ in the locus of intention, with the former serving to protect oneself, while the latter aims to protect others from potential risks. Chinese people not only worry for themselves but also have concern for their significant others over COIVD-19, which motivates IHB. Besides, home quarantine provided Chinese people with ample time and opportunity to communicate with and to influence their significant others (ie, engaging in IHB) both online and offline.

Both PHB and IHB contribute to curbing the spread of infectious diseases. On the one hand, PHB is self-serving, lowering one’s own vulnerability to a disease [10-12]. However, despite the health benefits of PHB, there exist variations in people’s adoption of preventive behaviors [5]. Hence, a close examination of the prevalence and potential correlates of people’s engagement in preventive behaviors toward COVID-19 is called for. On the other hand, IHB is other-serving, reducing others’ risks. A person’s active intervening health behaviors targeted at others may successfully persuade other people to adopt precautionary measures against the disease because, as social beings, people’s behaviors are subject to the influence of social relationships [13]. In essence, intervening behaviors can be treated as performing PHBs on behalf of others. Henceforth, we examine the influence of the same set of potential determinants on people’s engagement in PHB and IHB.

In particular, we employed the key components of the Preventive Health Model (PHM) [14]. PHM posits that people’s adoption of preventive behaviors are subject to the impacts of social influence (ie, social support and doctor-patient communication), psychological variables (ie, barriers, benefits, and self-efficacy of conducting precautionary behaviors), and program factors (eg, promotional communication or health information in media) [14]. All these factors were examined in our study. Additionally, we examined people’s media use behaviors. A considerable amount of research has found that media use of different platforms has an influence on people’s health behaviors [15-17]. Media literacy, defined as “the ability to access, analyze, evaluate, and create media in a variety of forms” [18], also has impact on people’s different health behaviors [19-21].

Apart from the previously mentioned psychographic variables, demographic variables have also been revealed to partially explain the variation in people’s propensity to adopt disease-related health behaviors [22]. As the Chinese population varies significantly along with demographics, we also included demographic variables in our investigation.

Ever since its outbreak in China, COVID-19 has seized national attention. China’s unprecedented and relentless efforts started to pay off in late March [1]. Unfortunately, the number of confirmed cases in other countries is rising [1]. The world’s fight against the coronavirus has just begun. Under these circumstances, investigating the Chinese public’s engagement in coronavirus-related health behaviors and identifying the psychological and demographic variables that are significantly associated with these behaviors are urgently called for. This paper aims to examine the demographic and psychological correlates of preventive and intervening behaviors during the outbreak of COVID-19, which could generate insights for implementing health interventions among the general public and helping with effective containment of COVID-19.

## Methods

### Recruitment

Using the service of a Chinese survey company, an online survey was distributed on different local social media platforms in China, such as WeChat and Baidu Post Bar, to access Mainland Chinese residents from February 2, 2020, to February 12, 2020, when COVID-19 began breaking out in China. The foci of the survey were to evaluate Chinese people’s propensity to engage in preventing health behaviors and IHBs, and to disentangle key determinants for people’s adoption of such protective measures. The study was approved by the Institutional Review Board at Lingnan (University) College, Sun Yat-sen University.

An electronic consent form was presented at the beginning of the survey. Only participants who were 18 years or older, currently located in China, and agreed to participate after reading the consent form were allowed to proceed in the survey. Respondents who completed the survey entered a lucky draw for a monetary incentive of around ¥6.00 (US $0.86).

### Measures

Our operationalizations of PHB and IHB followed the precaution behaviors that are recommended by the World Health Organization (WHO) for healthy people in responding to COVID-19 [23]. Specifically, *PHBs* were measured with five 5-point Likert scale (1=not at all, 5=very frequent) items that asked participants to report their frequency of engaging in the following behaviors: “wearing masks,” “washing hands,” “sanitizing clothes or other items,” “sneezing into their elbows,” and “staying at home” (α=.72). To assess participants’ engagement in coronavirus-related *IHBs*, we instructed participants to indicate whether or not they had persuaded their social others such as family and friends to “wear masks,” “wash hands,” “sanitize clothes or other items,” “staying at home,” and “sneeze into elbows” (dichotomous variables; 0=no, 1=yes).

Next, we assessed the potential psychosocial determinants for PHB and IHB: perceived barriers and benefits of taking preventive measures, self-efficacy, emotional support, and patient-centered communications. All variables were measured along 5-point scales, anchoring from 1 (strongly disagree or not at all) to 5 (strongly agree or very much).

We assessed participants’ perceived *barriers* (two items: “It is hard to buy masks” and “It is difficult to get sanitizers”; α=.84) and perceived *benefits* of preventive behaviors (two items: “Wearing face masks can help prevent the spread of the coronavirus” and “Using sanitizers can help prevent the spread of the coronavirus”; α=.87).

Following this, we evaluated participants’ *self-efficacy* by measuring their confidence at addressing the risk of COVID-19 (two questions: “How confident are you at your preventing behaviors toward the coronavirus?” and “How confident are you that you will not be infected with the coronavirus?” [24]; α=.86).

*Emotional support* was measured by one question on a 5-point Likert scale (1=strongly disagree, 5=strongly agree): “During the outbreak of the coronavirus, my friends or family have provided me with emotional support when I need it - such as talking over problems” (mean 4.01, SD 1.06) [25].

Afterwards, *patient-centered communication* was assessed by instructing participants to evaluate their previous experience with health care providers along with four items: “In general, my feelings were taken seriously,” “I was given a chance to ask all the health-related questions,” “My healthcare providers made sure I understand the things I needed to do to take care of my health,” and “My healthcare providers explained things in a way that I could understand” [26] (α=.94).

Additionally, *media literacy* was measured by four 5-point Likert scale items including “I look for more information before I believe something I see in messages,” “It is important to think twice about what messages say,” “I think about the purpose behind messages I see,” and “I think about the truthfulness of messages before I accept them as believable” (α=.84) [27]. One question was used to ask participants’ media use for COVID-19 news: “How frequently do you receive coronavirus-related news and/or information from the following media channels?” (1=never, 5=very frequent). *Social media use* was measured by four items: “Weibo,” “Wechat messages,” “Wechat public news accounts,” and “QQ messages or Qzone” (α=.62). *Traditional media use* was measured by three items: “TV,” “broadcast,” and “newspapers” (α=.76). Digital news media was measured by one item: “news app, news on websites or other format of news on the internet other than social media” (mean 3.30, SD 1.28).

Finally, participants provided their basic demographic information (age, gender, marital status, education background, and income level) and ended the survey. Details of scales used for variables under study are shown in Table 1.

**Table 1:** Measurement of study variables.

Variable and items		Cronbach α	Range
**Preventive behaviors: how frequently are you engaging in the following behaviors?**		.72	1-5
	Wearing masks		
	Washing hands		
	Sanitizing clothes or other items		
	Sneezing into your elbows		
	Staying at home (avoid going out)		
**Intervening behaviors: please indicate whether or not you have persuaded your social others such as families and friends to:^a^**		N/A^b^	0-5
	Wear masks		
	Wash hands		
	Sanitize clothes or other items		
	Stay at home (avoid going out)		
	Sneeze into elbows		
**Barriers**		.84	1-5
	It is hard to buy masks.		
	It is difficult to get sanitizers.		
**Benefits**		.87	1-5
	Wearing face masks can help prevent the spread of the coronavirus.		
	Using sanitizers can help prevent the spread of the coronavirus.		
**Self-efficacy**		.85	1-5
	How confident are you at your preventing behaviors toward the coronavirus?		
	How confident are you that you will not be infected with the coronavirus?		
**Emotional support**		N/A	1-5
	During the outbreak of coronavirus, my friends or family have provided me with emotional support when I need it, such as talking over problems.		
**Patient-centered communication**		.94	1-5
	In general, my feelings were taken seriously.		
	I was given a chance to ask all the health-related questions.		
	My health care providers made sure I understand the things I needed to do to take care of my health.		
	My health care providers explained things in a way that I could understand.		
**Media literacy**		.84	1-5
	I look for more information before I believe something I see in messages.		
	It is important to think twice about what messages say.		
	I think about the purpose behind messages I see.		
	I think about the truthfulness of messages before I accept them as believable.		
**Social media: how frequently do you receive coronavirus-related news or information from the following media channels?**		.62	1-5
	Weibo		
	Wechat messages		
	Wechat public news accounts		
	QQ messages or Qzone		
**Traditional media**		.76	1-5
	TV		
	Broadcast		
	Newspapers		
**Internet news channels other than social media**		N/A	1-5
	News apps, news on websites, or other format of news on the internet other than social media		

^a^The response was dichotomous.

^b^N/A: not applicable.

### Statistical Analysis

Since our hypotheses were developed based on the PHB model, we conducted two multiple regression analyses to examine the associations between the independent variables and the two behavioral outcomes. All analyses were performed in SPSS 25 (IBM Corp).

## Results

### User Statistics

A response rate of 55.30% (2980/5388) was obtained. A pretest of our survey with 7 volunteers revealed that the time spent on the questionnaire ranged from 5 to 31 minutes. Following the advice of the survey company and glancing over the answers, completed surveys that took less than 5 minutes were most likely invalid. Therefore, questionnaires that took less than 5 minutes were excluded from the analyses. Additionally, we excluded respondents who had missing data on key variables (independent and dependent variables) in this study. Since outliers of the data set may affect regression results [28], we dropped extreme data points using the explore function and checking the box plot; 2949 participants (18-85 years, mean age 31, SD 0.65 years) were included in the final analyses. Among all participants, 51.2% (n=1509) were female, 54.5% (n=1607) were married, 22.2% (n=656) had an annual household income of ¥100,000-¥150,000 (US $14,389-$21,584), and 49.8% (n=1467) had a college degree or above. Demographic information of the sample is shown in Table 2.

**Table 2:** Demographic and socioeconomic characteristics of the sample.

Characteristic			Participants (N=2949), n (%)
**Sex**			
	Male	1440 (48.8)	
	Female	1509 (51.2)	
**Age (years)**			
	18-24	786 (26.7)	
	25-29	549 (18.6)	
	30-34	653 (22.1)	
	35-39	477 (16.2)	
	40-44	189 (6.4)	
	45-49	138 (4.7)	
	50-54	79 (2.7)	
	55-59	53 (1.8)	
	≥60	25 (0.8)	
**Marital status**			
	Single	1342 (45.5)	
	Married	1607 (54.5)	
**Education**			
	High school graduate or less	727 (24.7)	
	Professional school	755 (25.6)	
	Bachelor’s degree	1131 (38.4)	
	Postgraduate degree	336 (11.4)	
**Income, ¥ (US $)**			
	<70,000 (10,072)	1030 (35.2)	
	70,001-100,000 (10,073-14,389)	519 (17.6)	
	100,001-150,000 (14,390-21,583)	656 (22.2)	
	150,001-300,000 (21,584-43,167)	479 (16.2)	
	>300,001 (43,168)	255 (8.6)	

### Evaluation Outcomes

#### Preventive Health Behaviors

**Figure 1:**
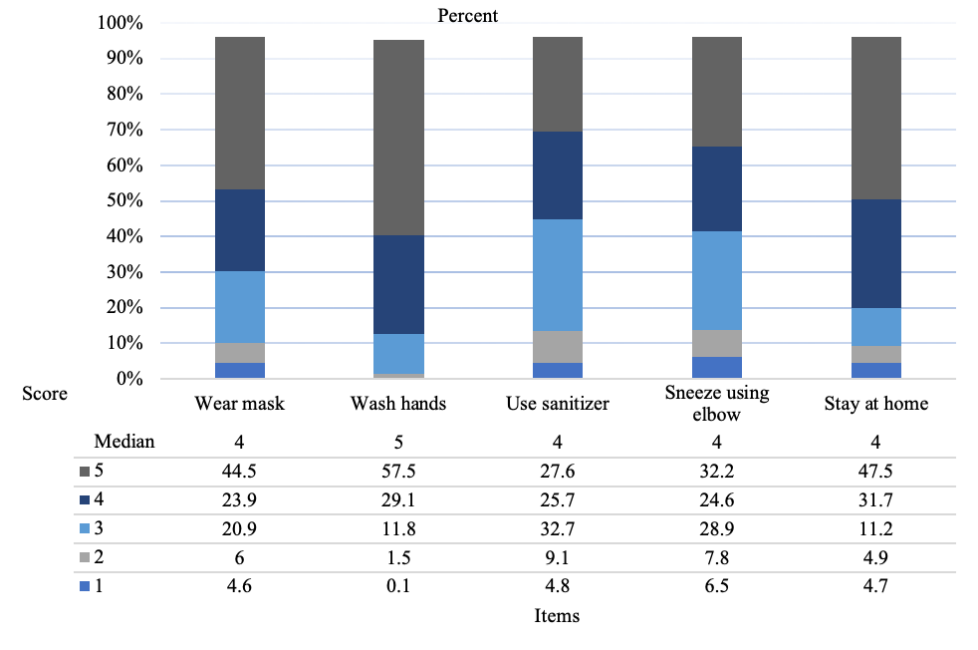
Frequencies of each preventive behavior.

On average, participants’ total frequency score of engaging in preventive behaviors had a mean of 4.00 (SD 0.65), with a possible range of 1 to 5. Of the 2949 respondents, approximately 55.3% of the participants reported frequent (ie, 4; n=935) or very frequent (ie, 5; n=694) engagement in preventive behaviors. Among the five preventive behaviors measured, the mean score for wearing a face mask was 3.98 (SD 1.14), washing hands had a mean of 4.42 (SD 0.76), using sanitizer had a mean of 3.62 (SD 1.12), sneezing into an elbow had a mean of 3.68 (SD 1.19), and not going out had a mean of 4.12 (SD 1.09). The distribution of each preventive behavior is shown in Figure 1.

A significant regression equation was found (*F*_14,2934_=31.07, *P*<.001; *R*^2^=0.13). Among the demographic predictors, only education level and marital status were significantly associated with preventive behaviors regarding COVID-19 (see Table 3). Individuals with higher education level (B=0.038, SE=0.012, *P*=.002) and those who were married (B=0.117, SE=0.030, *P*<.001) reported greater engagement in preventive behaviors. Among the psychosocial and behavioral predictors, perceived barriers and benefits of preventive behaviors; self-efficacy; emotional support; previous patient-centered communication with health providers; media literacy; and frequency of social media use, traditional media use, and internet news use other than social media for COVID-19 news were significantly associated with preventive behaviors. Individuals who reported fewer barriers of engaging in preventive behaviors (B=–0.055, SE=0.009, *P*<.001), higher benefits of the behaviors (B=0.098, SE=0.017, *P*<.001), greater self-efficacy (B=0.042, SE=0.014, *P*=.002), greater emotional support (B=0.031, SE=0.012, *P*=.01), greater previous patient-centered communication with health providers (B=0.029, SE=0.014, *P*=.04), higher media literacy (B=0.033, SE=0.016, *P*=.04), more frequent social media use (B=0.046, SE=0.016, *P*=.005), greater traditional media use (B=0.079, SE=0.013, *P*<.001), and greater use of internet news channels other than social media (B=0.038, SE=0.010, *P*<.001) for COVID-19 news engaged in greater preventive behaviors at the outbreak of COVID-19.

**Table 3:** Correlates of preventive behaviors among Chinese during the outbreak of the coronavirus disease.

Variable	Unstandardized coefficients		Standardized coefficients β	*P* value
	B	SE		
Sex	.024	.023	.018	.30
Age	.001	.002	.018	.44
Education	.038	.012	.063	.002
Marital status	.117	.030	.089	<.001
Income	.015	.008	.038	.06
Barriers	–.055	.009	–.104	<.001
Benefits	.098	.017	.109	<.001
Self-efficacy	.042	.014	.060	.002
Emotional support	.031	.012	.050	.01
Patient-centered communication	.029	.014	.042	.04
Media literacy	.033	.016	.043	.04
Social media	.046	.016	.057	.005
Traditional media	.079	.013	.129	<.001
Internet news channels other than social media	.038	.010	.074	<.001

#### Intervening Health Behaviors

The averaged index of IHB (mean 4.67, SD 0.77) revealed that overall participants engaged in more than four intervening behaviors out of the measured five behaviors. More than 97% (2864/2949) of the participants reported that they had ever advised others to wear masks, wash hands, and stay at home. Approximately 89.9% (2652/2949) of the participants had advised others to use sanitizer and 84.5% (2493/2949) had suggested others to sneeze into an elbow.

A significant regression equation was found (*F*_14,2934_=15.11, *P*<.001; *R*^2^=0.07). Income and marital status were significantly associated with people’s engagement in IHB (see Table 4). Particularly, individuals who were married (B=0.146, SE=0.037, *P*<.001) and had lower income (B=–0.040, SE=0.010, *P*<.001) reported greater engagement in IHB. That is, these people were more active in persuading other people to adopt protective measures against the disease.

Among the psychosocial and behavioral predictors, benefits of engaging in preventive behaviors; self-efficacy; previous patient-centered communication with health providers; media literacy; and frequency of social media use, traditional media use, and internet news channels other than social media for COVID-19 news had significant correlations with IHB. Particularly, individuals who reported greater benefits of engaging in preventive behaviors (B=0.052, SE=0.021, *P*=.01), greater self-efficacy (B=0.038, SE=0.017, *P*=.02), greater previous patient-centered communication with health providers (B=0.036, SE=0.017, *P*=.04), lower media literacy (B=–0.039, SE=0.019, *P*=.046), greater social media use (B=0.062, SE=0.020, *P*=.002), greater traditional media use (B=0.072, SE=0.016, *P*<.001), and greater use of internet news channels other than social media (B=0.039, SE=0.012, *P*=.002) for COVID-19 news were more engaged in performing IHBs.

**Table 4:** Correlates of intervening behaviors among Chinese during the outbreak of the coronavirus disease.

Variable	Unstandardized coefficients		Standardized coefficients β	*P* value
	B	SE		
Sex	0.026	0.028	.017	.35
Age	–0.001	0.002	–.018	.46
Education	0.014	0.015	.019	.36
Marital status	0.146	0.037	.095	<.001
Income	–0.040	0.010	–.086	<.001
Barriers	0.007	0.012	.011	.54
Benefits	0.052	0.021	.049	.01
Self-efficacy	0.038	0.017	.047	.02
Emotional support	0.011	0.015	.015	.47
Patient-centered communication	0.036	0.017	.045	.04
Media literacy	–0.039	0.019	–.043	.046
Social media	0.062	0.020	.066	.002
Traditional media	0.072	0.016	.099	<.001
Internet news channels other than social media	0.039	0.012	.065	.002

## Discussion

### Principal Results

Using a national online survey, we examined the potential predictors of two different types of epidemic-related health behaviors—the self-focused PHBs and the other-focused intervening behaviors—among Chinese people in the face of COVID-19 and pinned down key psychological determinants for Chinese public’s behavioral engagement. Our findings offer valuable implications that might be applicable to other regions with similar policies or cultures in attempts to encourage the general public’s adoption of precautionary measures.

Our surveyed participants’ reported locations covered most of the provinces and areas in China, and most of them reported highly active adoption of PHBs to protect themselves (1629/2949, 55.3% reported frequent or very frequent engagement) and of IHBs with the goal of protecting social others (2493/2949, 84.5% reported engagement in at least one of five behaviors). The mainland Chinese people’s active engagement in protective behaviors during the early outbreak of COVID-19 surely contributed to effective control of the epidemic. Mainland China’s daily new confirmed cases decreased from 2022 on February 11, 2020, (toward the end of survey distribution) to 21 on July 23, 2020.

Our attempts to find key factors that facilitate or debilitate participants’ propensity to take PHBs and IHBs revealed interesting findings. First, demographic variables have been found to exert differential effects on individuals’ adoption of protective measures. In 2015, around 75.34% of the Chinese population received an educational level of high school and above [29]. Therefore, we used high school or less as the reference group. Education level was found to be positively associated with PHBs, suggesting that individuals with higher education were more likely to engage in preventive behaviors to protect themselves, which is consistent with previous studies [30]. However, education level was not associated with people’s intervening behaviors. Regarding the null effect of education level on one’s engagement in IHB, we believe the reason lies in the other-oriented nature of IHBs, that is, persuading others to follow health measures to protect themselves. Hence, we believe this behavior is more likely to be affected by social factors or whether the individual has a significant other, such as one’s marital status and their interactions with important people in their life.

Interestingly, income was negatively associated with intervening behaviors, such that people with lower income have weaker other-oriented motivation than those with higher income. Said otherwise, faced with a health crisis, poorer participants are less likely to behave in other-serving manners, suggesting their greater self-focus. This finding might be attributed to the fact that low-income groups face more and tougher challenges in the face of a health crisis due to lack of critical resources such as health insurance [31]. Marital status was found to be significantly associated with both preventive behaviors and intervening behaviors. Specifically, married individuals engaged in more preventive behaviors and more behaviors that promoted other individual’s self-protection against COVID-19. This finding, in line with previous research [32,33], showcases health-related benefits of marriage. Marriage encourages adoption of healthy behaviors and motivates people to monitor, influence, and even control partners’ health conditions [32].

However, among married individuals, engagement in intervening behaviors could be potentially associated with more interactions with spouses, children, parents, relatives, and even friends. Future studies should further investigate and tease apart the differential influences of those different types of interaction on people’s engagement in intervening behaviors among married individuals.

Moreover, we observed interesting relationships between the examined psychological factors and participants’ engagement in PHB and IHB. On the one hand, we found negative association between barriers and participants’ engagement in PHB. Specifically, more perceived barriers deterred people’s adoption of PHB against COVID-19. We also found that greater benefits, self-efficacy in preventing COVID-19, and emotional support had positive relationships with adoption of PHBs, which are consistent with previous research [14,33]. On the other hand, we found greater engagement in IHB among participants who perceived higher benefits of preventing coronavirus and with a higher self-efficacy. Taken together, these findings suggest that communication with the general public on COVID-19 should highlight the benefits of health behaviors, reduce perceived barriers of taking actions, and enhance self-efficacy. Additionally, it can be beneficial to advise people to seek emotional support from close others in the face of COVID-19.

Further, it is found that participants who experienced high-quality patient-doctor communication prior to COVID-19 were more active in adopting precautionary behaviors and intervening behaviors. People who had high-quality interaction with doctors tend to build trust in precautionary measures that are recommended and hence have greater motivation to comply with these recommendations. These findings shed light on the benefits of building and maintaining good patient-doctor relationships in the face of public health emergencies.

This study also investigates the effects of media literacy and media use during an outbreak of an epidemic on health behaviors. Interestingly, media literacy was positively related to preventive behaviors and negatively associated with intervening behaviors. These findings suggested that individuals with a higher ability to distinguish media messages were more likely to engage in preventive behaviors for themselves. On the contrary, individuals with a lower ability to judge a media message or news related to COVID-19 tended to intervene more toward other people’s health behaviors [20,21]. Their trust of sentential or misinformation might potentially boost their intervening behaviors.

Media are usually the critical platforms to deliver news and health information, and could potentially contribute to the engagement of preventive or intervening behaviors. We found that more frequent use of both new media and traditional media for coronavirus news and information were associated with greater engagement in both preventive and intervening behaviors, indicating social media, traditional media, and internet news channels other than social media were effective platforms to disseminate COVID-19–related information to promote health behaviors. However, based on the values of coefficients, people used social media and traditional media more frequently to get information related to COVID-19 than internet news channels other than social media. Our study extends similar findings from a previous study conducted in the United States, which found general health information online was positively related to preventive behavior [34]. Our findings indicate the potential roles of social media and traditional media to deliver effective preventive campaigns related to COVID-19 [35,36].

### Limitations

We acknowledge several limitations of this study and point in directions for future research. The nature of cross-sectional survey data limited the causal relationships between variables being inferred. However, our findings are largely in alignment with previous research findings regarding health behaviors. Besides, a convenient sampling approach was used. Participants were largely those who owned a social media account or who had internet access, which may undermine the generalizability of the conclusions to the whole population of China. Yet, we believe this is an issue of less significance given the urgency of the issue and the commonality of risks imposed by COVID-19 on the general public. Future studies should use a probability-based sampling method to detect health behaviors regarding COVID-19 to generalize the findings. In addition, studies examining whether the digital divide has an impact on health behaviors are warranted. Although all measurements of this study were drawn from previous studies, validated scales such as different media use should be employed in future research. Besides, it should be noted that the variance explained by our model was relatively small, which suggests potential alternative predictors. Hence, future studies should examine other possible determinants using other relevant theories. Additionally, living alone or with others could also be associated with health behaviors. Future studies should take these into consideration. Moreover, in this paper, we did not examine potential mediators or moderators of the behavioral outcomes as some theories suggest. Finally, future studies should collect longitudinal data to examine the trends of people’s engagement in health-related behaviors as the epidemic develops and the mediators and moderators of both PHBs and IHBs.

### Conclusions

This study reveals that, during the early outbreak of COVID-19, Chinese people reported high engagement in preventive and intervening behaviors. Their compliance with the recommended health behaviors by the Chinese government and the WHO has alleviated the serious epidemic and resulted in a controlled situation in March 2019. This study demonstrates the associations of psychosocial factors including the perceived barriers and benefits of health behaviors, self-efficacy, emotional support, patient-centered communication, media literacy and media use for COVID-19 news, and demographic factors such as education, income, and marital status with individuals’ adoption of health behaviors. Our findings have practical implications for policy makers and health organizations to design more effective health intervention programs using different media channels.

## Abbreviations

COVID-19: coronavirus disease
IHB: intervening health behavior
PHB: preventive health behavior
PHM: Preventive Health Model
WHO: World Health Organization
